# How to improve the mechanical safety of a novel spinal implant while saving costs and time

**DOI:** 10.1002/jsp2.70026

**Published:** 2024-12-25

**Authors:** Annette Kienle, Hans‐Joachim Wilke, Christian Schröder, Andrea Pietsch

**Affiliations:** ^1^ SpineServ GmbH & Co. KG Ulm Germany; ^2^ Institute of Orthopaedic Research and Biomechanics University Hospital Ulm Ulm Germany; ^3^ TÜV Süd Product Service GmbH Hamburg Germany

**Keywords:** approval, biomechanical testing, implant, mechanical safety, mechanical testing, performance, predicate device, risk‐analysis, spine

## Abstract

**Background:**

Spinal implant failure is associated with prolonged patient suffering, high costs for the medical device industry, and a high economic burden for the health care system. Pre‐clinical mechanical testing has great potential to reduce the risk of such failure. However, there are no binding regulations for planning and interpretation of mechanical testing. Therefore, different strategies exist. Mainly for novel implants an option is to start with a structured scientific literature search that forms an objective background for the definition of an implant‐specific test plan, the derivation of acceptance criteria and interpretation of the test results.

**Methods:**

This paper describes, how a literature‐based approach can look like from the initial literature search through the derivation of the test plan and the acceptance criteria, to the final test result evaluation and how this approach can support the proof that the device meets all necessary safety and performance standards.

**Results:**

The main advantage of this literature‐based approach is that testing and test result interpretation are linked with the loads acting on the individual implant in vivo. In an ideal case, testing is focused on the individual implant in a way that ensures maximum efficiency during the development and approval process combined with maximum insight in safety and effectiveness of the implant. Even comparative implant testing may become obsolete, which is a big advantage if comparative implant and related data are not available.

**Conclusion:**

This approach to pre‐clinical mechanical testing offers the potential to create a chain of arguments, from literature review through testing to the interpretation of test results. This methodology can significantly enhance testing efficiency, reduce risk of failure, and ultimately prevent unnecessary patient suffering and healthcare costs. By synthesizing scientific insights with regulatory requirements, this review aims to guide clinicians and researchers in improving patient care and advancing device technologies.

## Graphical abstract

This manuscript shows how the scientific literature can be used to proof that a novel spinal implant meets all necessary safety and performance standards for clinical application. The scientific literature has gained more and more importance for this purpose over the last few years, but still a structured approach from literature to implant approval has not yet been described anywhere so far. This work aims to close the gap between science and practical application in this field.

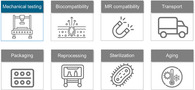

## 1 INTRODUCTION

Implant failure has a significant human and economic impact. The cost of such failure to the medical device industry was reported to range between 2.5 and 5 billion USD per year on average.^1^ Williamson et al.^2^ reported an increase of the cost of surgical intervention divided by the life years gained by a factor of two if there is any complication.

Spinal implant failure bears a particularly high risk of serious clinical complications due to the anatomical proximity of the spine to the spinal cord and the nerve roots. Depending on factors such as the type of implant or the spinal area, the reoperation rates due to hardware failure vary between less than 0.5% to more than 60% (Tables 1, 2, 3).

**TABLE 1:** Examples of failed implants. Spinal deformity correction: Rod breakage^3^; Motion preserving extradiscal implants: Wear^4^; Artificial disc replacement: Subsidence and retropulsion.^5^

Spinal deformity correction	Motion preserving extradiscal implants	Artificial disc replacement
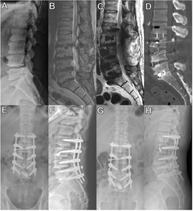	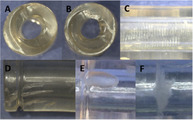	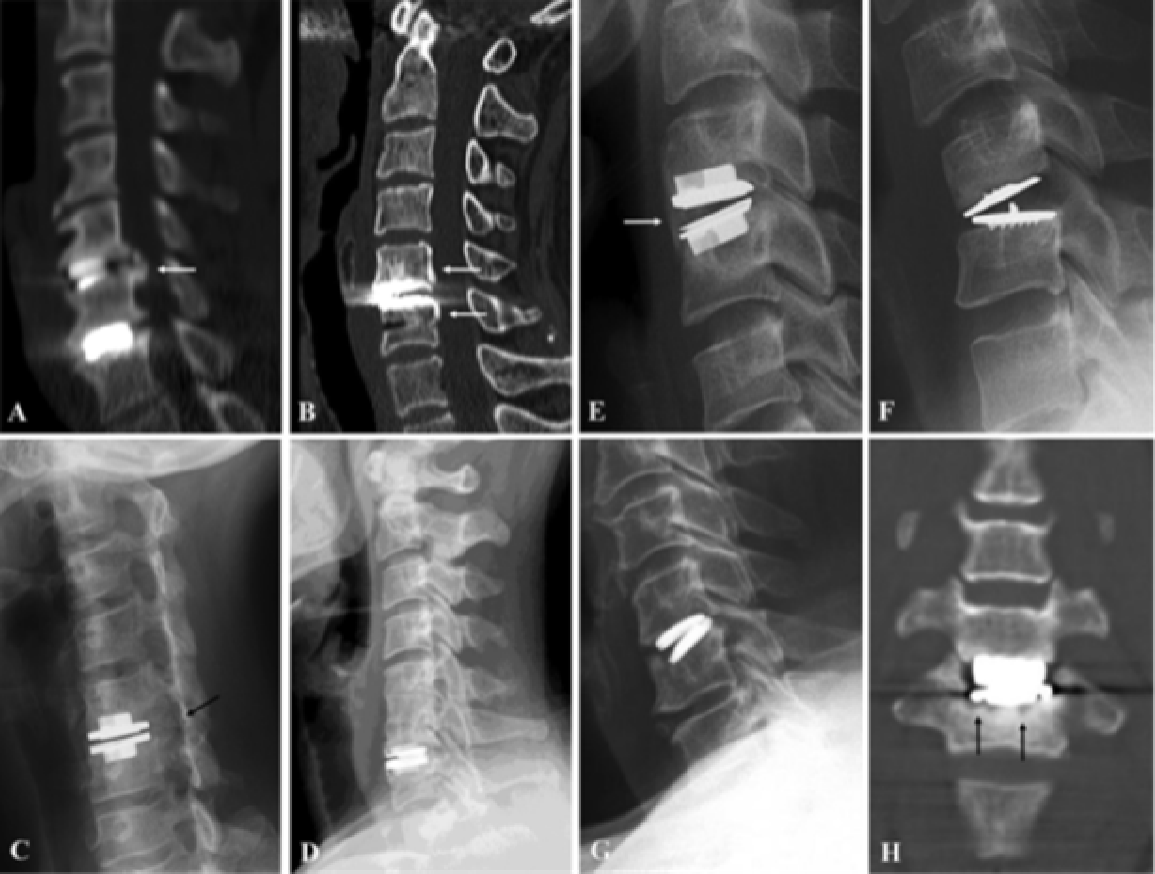

**TABLE 2:** Representative implant failure rates for cervical spine surgery as reported in literature.

Treatment	Complication rates cervical spine
Cervical spine surgery in general^6^	Reoperation rate 1.24% (30 days after surgery) and 3.30% (90 days after surgery) Out of those, 4% (30 days) and 6% (90 days) because of hardware failure
Cervical disc replacement^5^	Subsidence rate of 29% and osteolysis rate around the implant of 14%

**TABLE 3:** Representative implant failure rates for lumbar spine surgery as reported in literature.

Treatment	Complication rates lumbar spine
Non‐segmental posterior lumbar fusion^7^	Hardware failure rate 2.7% and the revision rate was 4.1% (out of 108 137 patients)
Lumbar deformity correction^3^	2.6% hardware failures (mean follow‐up of 1182 days)
Dynamic pedicle screw system (Dynesys)^8^	Case report of a patient who experienced pedicle screw breakage
Dynamic pedicle screw system (Dynesys)^4^	Polymer wear debris and an associated foreign‐body macrophage response in several cases
Two different dynamic fixators^9^	Hardware failure rate 11% and 64%
Charite Disc Prosthesis^10^	Inflammatory reaction in the periprosthetic fibrous tissue in 5 out of 5 cases
Lumbar nucleus replacement devices^11^	Retrieval incidence 48.8% (9 years after surgery) Main causes: significant loss of disk height at the operated level, displacement, silicone inside the spinal canal, migration

Based on these findings, it is obvious that effective measures must be taken to further reduce the risk of implant failure in those implant groups where the failure rates are high and to keep them low where they are already small. This puts pre‐clinical mechanical testing into the center of interest.

However, planning and interpretation of mechanical implant testing do not adhere to any binding rules. Therefore, any convincing procedure may be applied. Most commonly, either standard test methods are applied, and the results are compared to those of a predicate device, or a risk‐ or literature‐based approach is followed (Table 4). In any case, an appropriate justification is required.

**TABLE 4:** Two possible approaches to derive a pre‐clinical mechanical test plan, which can also be applied for a portfolio extension. Both approaches may also be combined.

Approach	Description	Advantages	Disadvantages
Standard test methods	Search for applicable test standards, screen Guidance Documents, etc.	Established and standardized test methods Possibly comparative data available (benchmark data)	No or only sparse reference to the load acting on the implant in vivo No in vivo‐based risk analysis possible For novel implants potentially incomplete
Literature‐ or risk‐based approach	Search for the loads acting of the implant in vivo (both, related to the surgical technique and related to the situation after implantation) and derive a test plan thereof	Test plan reflects loads acing on the implant in vivo	Scientific literature database has gaps, assumptions are to be made For standard implants possibly more detailed than necessary
Combination of the above	Search for the loads acting of the implant in vivo and derive a test plan and/or acceptance criteria thereof, then search for test standards, Guidance Documents, etc. that match the above test plan and adjust these standard test methods where required	Test plan reflects loads acing on the implant in vivo Established and standardized test methods wherever possible Possibly comparative data available (benchmark data)	For standard implants possibly more detailed than necessary

Finite element analysis (FEA) or any other numerical simulation method can support mechanical testing in complementary area such as the derivation of the worst‐case‐sizes and designs. However, simulation does not replace mechanical testing in most key areas such as fatigue, wear, or corrosion testing.

Similar to the derivation of the test plan, acceptance criteria can also be defined following different approaches (Table 5).

**TABLE 5:** Approaches to derive acceptance criteria for pre‐clinical mechanical testing. Both approaches may also be combined.

Approach	Advantages	Advantages	Disadvantages
Predicate device	Repeat testing with a suitable predicate device	Direct comparison with a clinically successful device	Often not possible in case of novel implants Possibly not fully comparable due to differences in design, intended purpose, material, or manufacturing Can end up with an oversized implant in case the predicate device is better than required Difficult to purchase
In vivo loading	Derive acceptance criteria from the in vivo loading	In vivo based Can be made available for all implants whether standard or novel	Scientific literature database has gaps, assumptions are to be made
Combination of the above	Repeat testing with a suitable predicate device, then, wherever, there are gaps, derive acceptance criteria from the in vivo loading	Direct comparison with a clinically successful device Wherever required (to fill gaps, to prevent oversizing, etc.) in vivo based criteria	None

All the above approaches, if thoroughly applied, have the potential to deliver a full chain of arguments towards the implant's mechanical safety.

The decision, which one to use is a case‐by‐case decision. For example, for a novel implant it is mostly recommended to focus on the literature‐ or risk‐based approach, whereas a standard implant may get by if tested according to standard test methods and compared to a predicate device.

Standard test methods have been applied and the results compared to predicate devices for several decades of years. Accordingly, in the medical device community, the level of awareness of this procedure is high.

This is not the case for the literature‐ or risk‐based approach. For that reason, to help people becoming more familiar with this approach, the aim of this paper is to provide a step‐by‐step description and discussion from the initial literature search through test plan derivation to test result evaluation.

## 2 MATERIALS AND METHODS

Three structured literature searches according to MEDDEV 2.7.1 rev 4 were carried out to objectively illustrate the scientific rationale behind the literature‐based approach to mechanical implant safety.

The topics of these literature searches were:

- ‘Search 1, hardware failure’: Frequency of spinal implant hardware failure
- ‘Search 2, functional testing’: State‐of‐the art in functional in vitro testing of spinal implants
- ‘Search 3, standard testing’: State‐of‐the art in standard testing of spinal implant

In all three cases, a database literature search was conducted online in English through the PubMed, Scopus and ScienceDirect databases for each of the three searches separately based on the following key‐words (Table 6):

**TABLE 6:** Key‐words used for the three literature searches.

Hardware failure	Functional testing	Standard testing
Spine	Spine	Spine
Implant	Implant	Preclinical
Hardware	In vitro	Testing
Failure	Range of motion	ISO
[Document type: review]	[Years: 2015–2024]	ASTM
[Subject area: medicine]		[Subject areas excluded: chemical engineering, materials science]

Before starting the literature search, exclusion criteria were defined (Table 7).

**TABLE 7:** Exclusion criteria used for the two literature searches.

Hardware failure	Functional testing	Standard testing
Studies not available in English and German	Studies not available in English and German	Studies not available in English and German
Studies where no abstract or full‐text is available	Studies where no abstract or full‐text is available	Studies where no abstract or full‐text is available
Studies regarding failures other than hardware failure	Animal studies	Animal studies
Scoliosis or adult deformity treatment Malpractice	In silico studies Studies that are not primarily focused on spinal implants	Studies not based on the pertinent international testing standards

After each single search, the number of search results was noted. Then, based on the titles and abstracts of all search results, those publications, favorable and unfavorable, were selected, that did not meet the exclusion criteria. After a cross‐check for duplicates, the full‐text of these potentially relevant publications was screened to select the ultimately relevant articles according to the above‐mentioned exclusion criteria. These were then passed over to analysis.

In addition to the above database search, literature cited in the relevant publications was screened and the whole history was summarized in the search statistics (Table 8).

**TABLE 8:** Search statistics of the implant failure, standard testing, and functional testing literature searches.

Step	Implant failure	Functional testing	Standard testing
Records identified through database search	33	18	13
Records identified through other sources	2	0	0
Records after duplicates removed	26	18	13
	**Screening**		
Records removed after screening title, abstract, and full‐text	24	9	4
Crosslinked records	3	6	0
Potentially relevant	2	9	9
Appraisal	5	15	9
	**Appraisal**		
Ultimately relevant	5	15	9
	**Analysis**		

## 3 RESULTS

The results of the present literature search are meant to improve the understanding of the risk‐ and literature‐based approach used to proof the safety and performance of a medical device. This approach includes the following steps:

1. Risk analysis and design input and output
2. In vivo loading of the implant
3. Test plan – supported by the structured literature searches regarding implant failure, standard testing, and functional testing
4. Acceptance criteria
5. Worst case sizes and designs
6. Testing
7. Assessment of the results

### 3.1 Risk analysis and design input and output

A risk analysis based on the ISO 14971 standard is required to identify and evaluate the risks that are associated with the use of an implant. It is first issued with the original project idea and is continuously revised throughout the whole product development lifecycle and design transfer. The risk analysis goes hand in hand with the documentation for design input and output, which is an important part of the Design History File to demonstrate that the device is safe and effective for its intended use.

The risk analysis is the core document that delivers arguments required to decide whether:

- testing is not required because the risk can be excluded or is addressed elsewhere,
- testing may be required (usually for innovative designs and new technologies), or
- the risk can be acceptable through predicate testing (if risk and benefit are comparable to the predicate device).

At that point, the question is how to address all those risks, where testing may be required:

- How should testing look like?
- What are the acceptance criteria?
- How can the right conclusions be drawn from the test results?

The literature search conducted regarding hardware failure (‘Search 1, hardware failure’) gives some insight in how mechanical risks can be identified and evaluated in the risk analysis. The results of the structured literature research were grouped based on the implant type:

- Internal fixators:
- Pressman et al.^3^ investigated factors associated with hardware failure after lateral thoracolumbar fusions. In summary, 6 out of 232 patients (2.6%) developed hardware failure (rod fracture, screw pullout, or screw breakage). Greater levels of posterior fusion, and greater numbers of interbody devices were associated with higher rates of hardware failure.
- Anterior plating:
  The clinical study reported by Stulik et al.^12^ included a study group with a dynamic anterior cervical plate and a control groups with a rigid anterior cervical plate. In the control group four patients (6.4%) demonstrated surgical hardware complications (1× plate breakage; 2× lower screws dislocation through the vertebra, 1× lower surgical screw back‐out). In the group with dynamic plate such failure was not observed. Thus, in this case, dynamic fixation was superior to rigid fixation.
- Motion preserving spinal implants:
  In the study reported by Oikonomidis et al.,^9^ patients were treated due to degenerative disease of the lumbar spine or spondylolisthesis with lumbar interbody fusion and dynamic stabilization of the cranial adjacent level. This clinical study was stopped as the hardware failure rates became too high with 5× fatigue fracture of the dynamic rod in one test group (11%) and 7× material failure of the dynamic part in the other test group (64%).
  Similar to the data from Oikonomidis et al.,^9^ other types of motion preserving implants also caused relatively high failure rates. van Ooij et al.^10^ investigated the polyethylene wear debris and long‐term clinical failure of the Charité Disc Prosthesis. 4 patients underwent anterior lumbar revision due to failure of total disc replacement surgery. All of the retrieved polyethylene cores showed evidence of wear, but the extent and severity varied among the 4 patients. In 3 of the 4 patients, implant wear was associated with an unfavorable biomechanical environment (e.g., subsidence, migration, undersizing, and adjacent fusion).
  In a clinical series of 125 patients treated with nuclear replacement devices after 9 years follow‐up, the overall retrieval incidence was 48.8%.^11^ Endplate reactions, subsidence and expulsion occur in a high percentage of patients over time.

In summary, according to this search the following points are among those that should be considered when conducting a risk analysis:

- Length and composition of a spinal construct can influence the risk of failure
- Flexibility of a spinal implant should be addressed regarding the risk of failure at the screw‐bone anchorage
- Any unfavorable biomechanical environment (incorrect size selection, incorrect placement, etc.) should be considered
- Use time and lifetime in the body should be acknowledged (e.g., corrosion, material aging, fatigue)

### 3.2 In vivo loading of the implant

**FIGURE 1:**
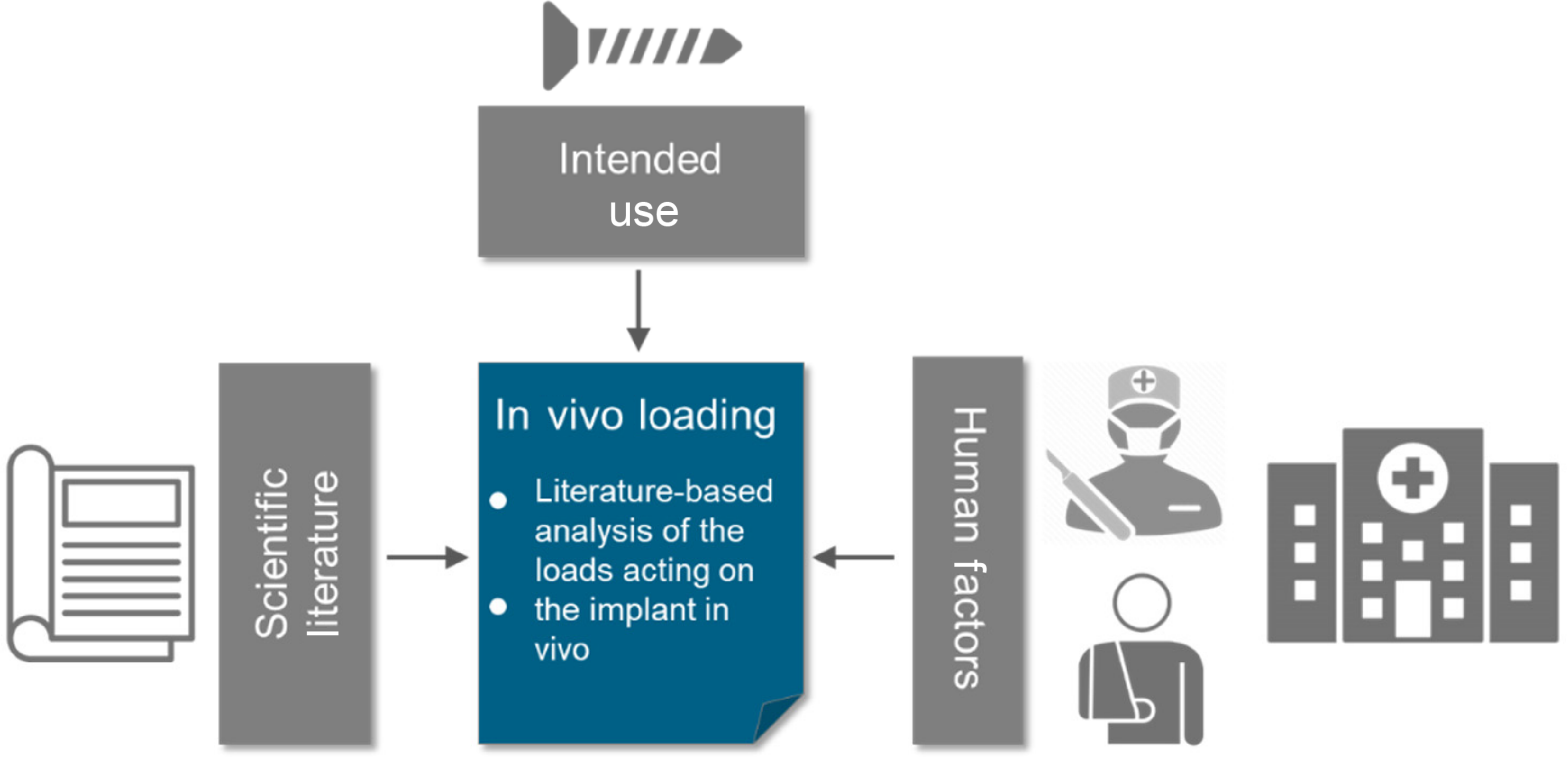
Step 1 of the literature‐based approach to proof the mechanical safety of a spinal implant: Summary of the in vivo loading of the relevant spinal region and the implant under consideration of the intended use of the implant and human factors.

According to the literature‐based approach, wherever testing may be required, a literature database is first established. This database is built up through a scientific literature search using established databases such as PubMed or Scopus. In an ideal case, the results of this literature search cover all loading patterns and the loading amplitudes acting on the implant after surgery and specifically addresses the skeletal region to be treated, the intended use of the implant and human factors (Figure 1).

In general, the most important questions to be answered are:

- Different spinal regions and the transition zones in between experience different loading patterns. Which load components are acting on the specific spinal region in vivo?
- Is there any load sharing between the implant and remaining anatomical structures?
- The spine has six degrees of freedom, thus, possibly all three force and three bending moment components may have to be considered. Are all these load components relevant for the specific implant?
- Do the relevant load components act simultaneously on the implant?
- What are the loading amplitudes in vivo during single‐cycle extreme or repetitive everyday activities?
- What are the human factors, that are specifically relevant for the implant?

Different literature sources may be appropriate to answer these questions such as in vitro studies on cadaveric specimens, in vivo studies on volunteers or in silico studies.

Human factors are increasingly gaining attention when it comes to the mechanical safety of implants. This trend, however, is not yet reflected by the scientific literature. There are only few human factors studies published that relate to the mechanical safety of spinal implants. Drazin et al.^13^ reported that the navigation performance in spine surgery improves with the experience level. Also, Vieweg^14^ mentioned the importance of technical skills and non‐technical (social competence) skills of the staff regarding complication minimization.

**FIGURE 2:**
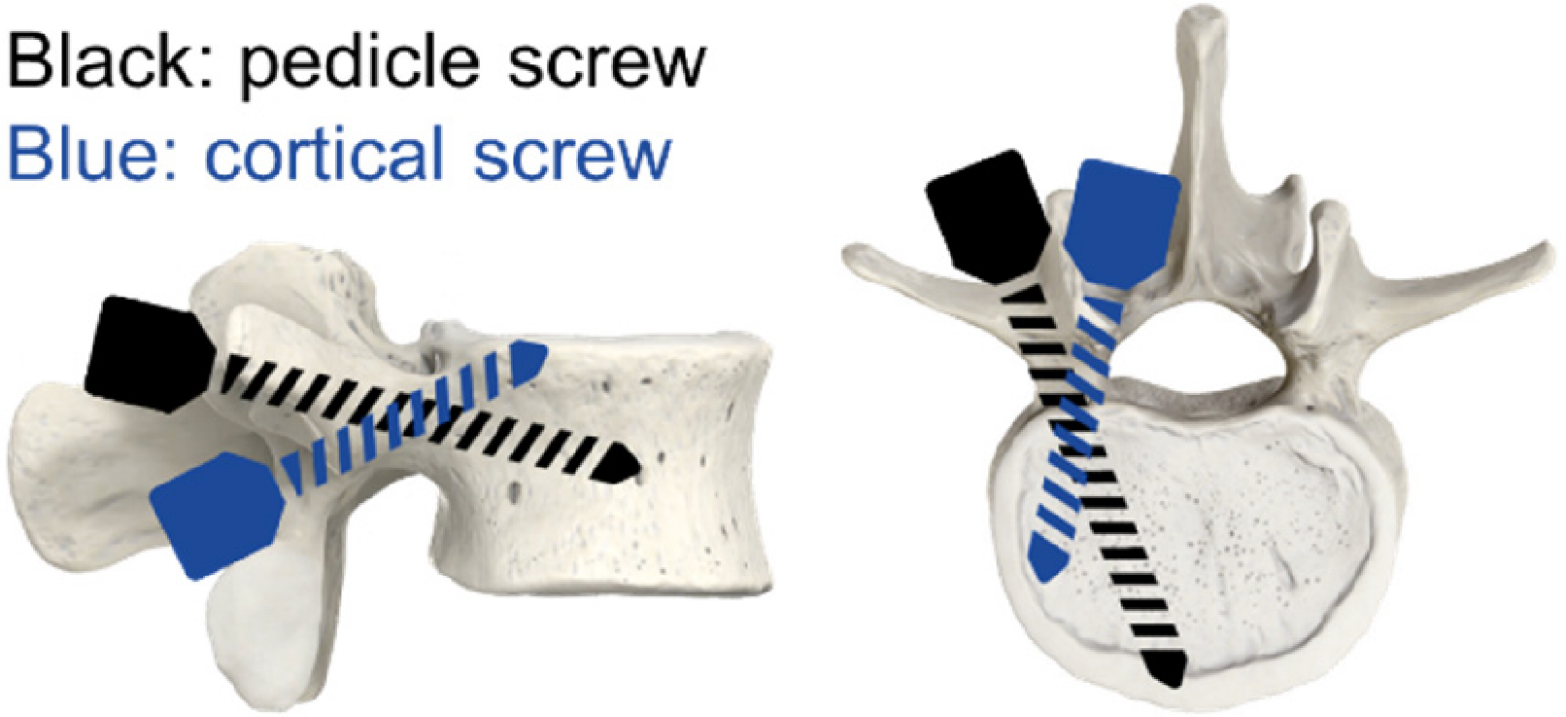
Examples of two different screw trajectories: Pedicle screw (black), cortical screw (blue). Modified according to Verma et al.^15^ There are different preferences among surgeons, however, whether these preferences have any effect on mechanical safety has not yet been clarified.

Other studies show that surgeons may prefer different implantation procedures. Verma et al.^15^ for example reviewed screw fixation options available for treating lumbar instability, degeneration, and deformity. Focus was put on common and emerging screw fixation techniques in the lumbar spine (Figure 2). Whether any of these insertion techniques has a certain impact on mechanical stability has not been investigated but care should be taken during pre‐clinical testing to cover such variation.

**FIGURE 3:**
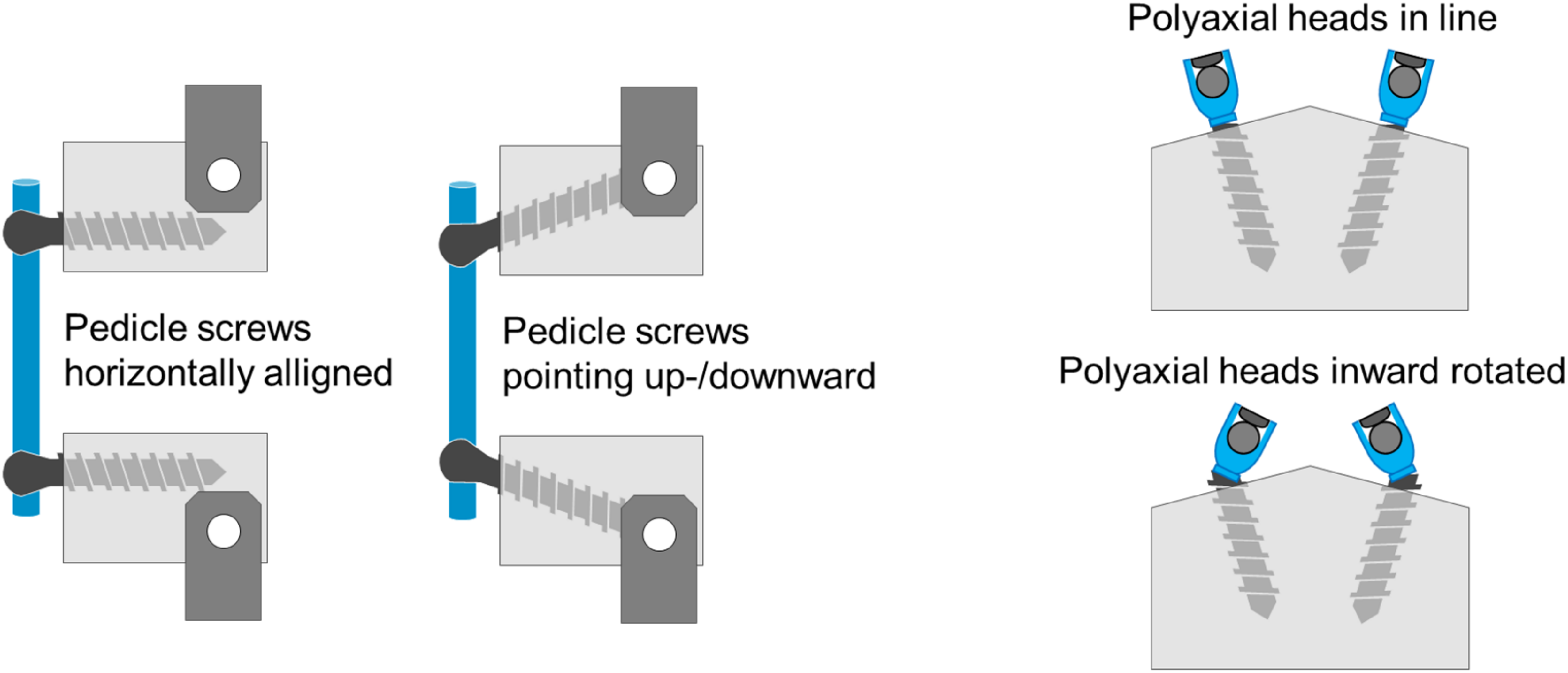
Examples how human factors could be addressed in pedicle screw testing according to the ASTM F1717 standard. Left: Screw orientation horizontal (standard) versus upward/downward pointing; Right: Polyaxial screw in line with screw axis (standard) or inward rotated.

Similarly, Noriega et al.^16^ investigated the whether the interfacing angle between pedicle screws and rods affect clinical outcomes after posterior thoracolumbar fusion. They found that this angle possibly influences the stresses in the implants. Also, the screw‐bone interface may be affected. Çetin et al.^17^ found that angled insertion of the pedicle screws significantly decreased the pullout stiffness in all diameters (Figure 3).

These human factors may either be addressed using a literature‐based path or by inclusion of specific tests in the test plan.

### 3.3 Test plan

Now as the in vivo loading is known from step (2), the test plan can be established. The test plan is the core‐document of the risk‐ and literature‐based approach. It describes all mechanical tests that are to be carried out.

**FIGURE 4:**
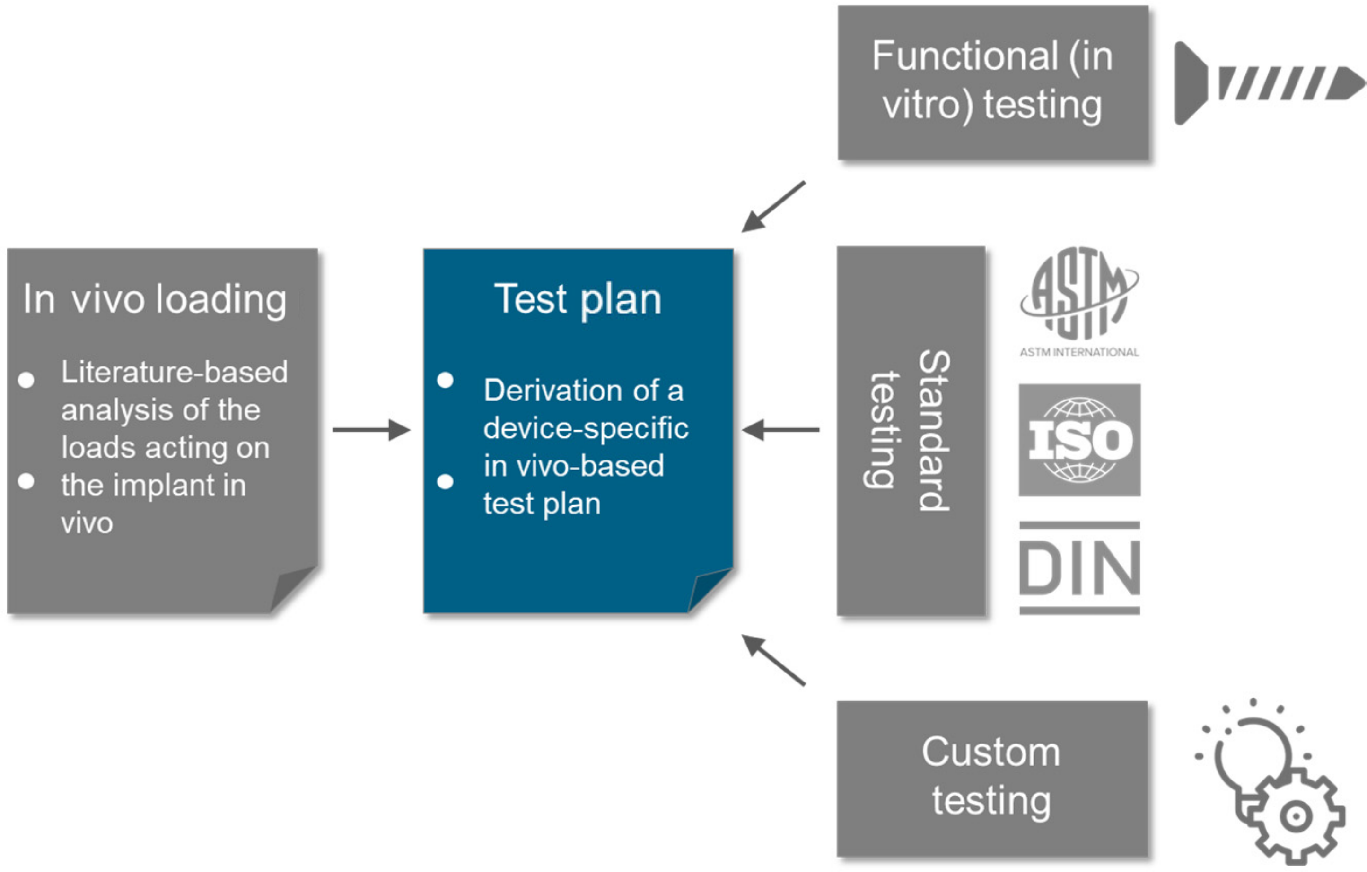
Step 2 of the literature‐based approach to proof the mechanical safety of a spinal implant: Derivation of a mechanical test plan based on the in vivo loading conditions and taking into consideration the specific function of the implant, available test standards and custom test methods.

The derivation is based on the in vivo loading of the implant. Furthermore, the risk of implant failure, the specific function of the implant, standard test methods and custom test methods must be considered (Figure 4). In case there is enough evidence from literature that testing is not required, this argumentation is to be provided instead.

When drawing up the test plan, the significance of finite element analyses (FEA) is coming into discussion. Currently. FEA is a well‐established tool for the derivation of the worst‐case sizes and geometries for testing – especially in cases, where a theoretical argumentation reaches its limits. In contrast, FEA is not used as a substitute for physical testing – especially not in cases where fatigue, wear, 3D‐printed porous structures, or coated materials are of interest. Thus, to date, FEA supports physical testing but does not replace it.

#### 3.3.1 Functional testing

To give an idea of how functional in vitro testing can look like, the results of the structured literature search ‘Search 2, functional testing’ are presented in the following.

Functional testing differs from standard mechanical testing at several point:

- Functional testing is focused on the function that an implant is intended to perform. For example, in case an implant aims to restore part of the movement of a spinal segment, it may be helpful to investigate whether it can actually do so.
- Functional testing is mostly carried out in vitro using cadaveric specimens, as testing requires real spinal functional units.
- Functional testing is restricted to static or short cyclic testing, as cadaveric specimens do not allow for long‐term testing.

The structured literature search showed that testing mostly aims at the following outcome parameters:

- Range of motion^18^, ^19^, ^20^, ^21^, ^22^, ^23^, ^24^, ^25^, ^26^:
  Spinal segment or adjacent segment flexibility after treatment is of importance for both, implants for rigid fixation as well as implants that aim to preserve full or part of the physiological motion.
  For the measurement of the range of motion of a spinal segment in vitro testing criteria were recommended by Wilke et al.^27^
- Center of rotation, helical axes, coupled motions^19^, ^23^, ^24^:
  For motion preserving implants motion characterization is highly important as the kinematics of the healthy spine has to be restored. Otherwise, treatment may cause unphysiological loading that possibly results in secondary diseases.
  The advantages of helical axis against range of motion were discussed by Wilke et al.^28^
- Intradiscal pressure, facet joint forces^26^:
  These parameters help to identify the effect of a certain treatment on adjacent structures such the intervertebral disc or the facet joints. A better understanding of these loads can prevent secondary diseases related to overloading or unphysiological loading of these structures.
- Screw loosening^19^, ^29^, ^30^:
  In vitro tests may be a good alternative to investigate the implant bone interface as real bone behaves differently compared to standardized bone replicate material such as polyurethane foam.
- Cyclic subsidence or extrusion/expulsion^31^, ^32^:
  Cyclic testing in vitro is less common as it is technically more demanding and limited in duration by the degradation process of the cadaveric specimens. But still, it can help to investigate the behavior of an implant under repetitive load application such as subsidence of cages or extrusion of nucleus implants.

#### 3.3.2 Standard testing

Standard test methods are mostly well established, but it sometimes remains unclear how a certain test method refers to a certain situation in vivo. In case a standard test method seems reasonable, but a certain parameter should be modified based in the derived in vivo loading, modification is required.

In the structured literature search ‘Search 3, standard testing’, three scientific studies were found with a link to at least one standard testing procedure: Arnin et al.^33^ investigated a minimal invasive deformity correction system for patients with adult lumbar scoliosis. Among others fatigue loading was adapted to the ASTM F1717^34^ standard. Fogel et al.^35^ even followed two different standards for intervertebral body fusion cage^36^, ^37^ and Wu et al.^38^ determined the wear behavior in a cervical total disk replacement system based on the ISO 18192‐1^39^ standard. However, all in all, standard testing can only rarely be found in the scientific literature.

In a series of publications La Barbera and colleagues investigated the ASTM F1717^34^ and ISO 12189^40^ standards.^41^, ^42^, ^43^, ^44^, ^45^, ^46^, ^47^ This data is meant to provide information to better understand the advantages and limitations of these standard test methods and their in vivo correlation.

In these studies it was shown that the ASTM F1717^34^ standard prescribes geometric data that does not necessarily represent a reasonable worst case as a comparative parametric investigation demonstrated a significant (up to a 22.2%) increase in the stress on the device, compared to the standard that is currently in use.

Regarding the ISO 12189^40^ standard, the initial precompression was shown to be important regarding the stresses experienced by the rods whereas the anterior support stiffness seemed to play the most detrimental effect on the loads acting on the implant. When combining the effect of anterior support stiffness with the initial precompression, stress variations beyond 427% were be reached.

In summary, this search revealed the following critical points to be considered during preclinical standard testing:

- Standards do not necessarily represent a reasonable worst‐case. They may have to be modified to meet the requirements of a specific implant.
- Standards do not always prescribe all boundary conditions in detail. Wherever there is room for variation well‐founded decisions must be made and documented.

In addition to the above points, there is often debate about the sample size, which is the number of samples per test that allows for statistical analysis. The ideal sample size depends on many factors such as the risk class or the standard deviation of the single test results. It is therefore recommended to find arguments in favor of each selected sample size – even in cases where recommendations are made in the test standards. Other standards or draft standards from different testing areas may be used as a theoretical background.^48^, ^49^

### 3.4 Acceptance criteria

**FIGURE 5:**
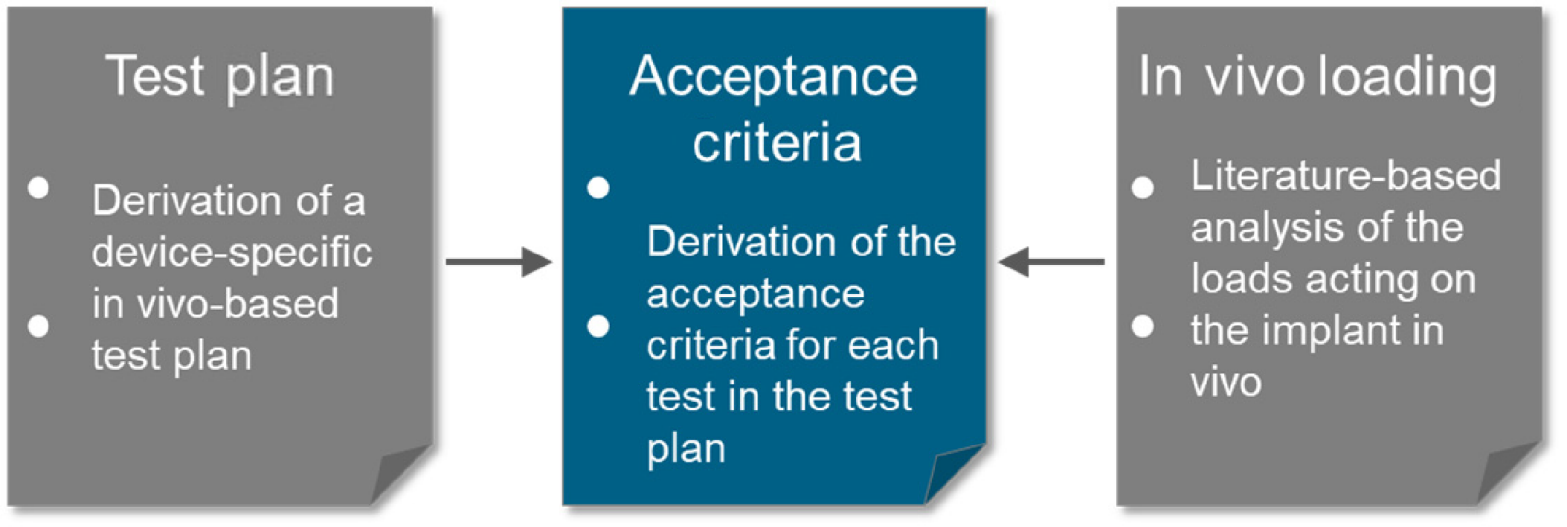
Step 3 of the literature‐based approach to proof the mechanical safety of a spinal implant: Derivation of acceptance criteria for each single test described in the test plan. This derivation is based on the in vivo loading as summarized in step 1.

For each test described in the test plan, acceptance criteria are required. In case the literature‐based approach is followed, and the scientific literature database already exists, the derivation of the acceptance criteria is straight forward (Figure 5).

The acceptance criteria are derived based on the in vivo loading for each test mentioned in the test plan separately. Derivation of the acceptance criteria should also consider differences in the load application rate (static versus dynamic fatigue testing) and different outcome parameters. Fatigue testing, for example, is interesting in terms of the fatigue load but may also be interesting in terms of the wear rate.

As one can imagine, the scientific literature database always leaves some room for interpretation. A well‐established procedure in this case is to follow realistic worst‐case strategy.

### 3.5 Worst case sizes and designs

**FIGURE 6:**
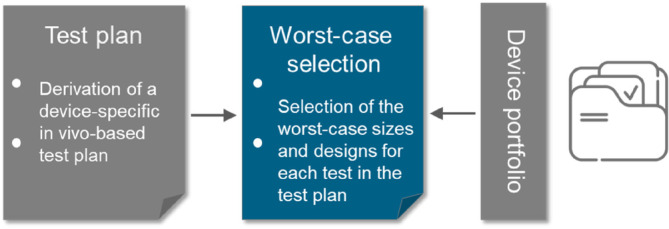
Step 4 of the literature‐based approach to proof the mechanical safety of a spinal implant: Selection of the worst‐case sizes and designs of the implant for each single test described in the test plan.

The worst‐case sizes and designs of the implant depend on the device portfolio and should be defined for each single test separately (Figure 6).

It is important to take care of all implant‐specific features that could have an influence on its stability under the respective loading conditions. This argumentation may be done based on numerical simulation (FEA) and/or based on a geometry‐ and material‐related justification.

### 3.6 Testing

As soon as the above documentation is finalized, mechanical testing can be conducted as defined in the test plan.

As described above, beyond standard testing, modified standard testing or testing according to individual test procedures may be required such as:

- Impact tests for implants made of a brittle material or implants with coatings that experience sudden loads during implantation (impaction of implants) or after surgery (certain activities of the patient)
- Wear and corrosion tests in case of implants with interfaces between implant components or implants that are made of material combinations.

Care should be taken to justify each single test parameter for example based on the above literature search. Also, verification and validation of the procedure should be provided before application.

In contrast to worst‐case derivation, FEA is less common to replace testing. Especially for fatigue and wear testing.

### 3.7 Assessment of the results

**FIGURE 7:**
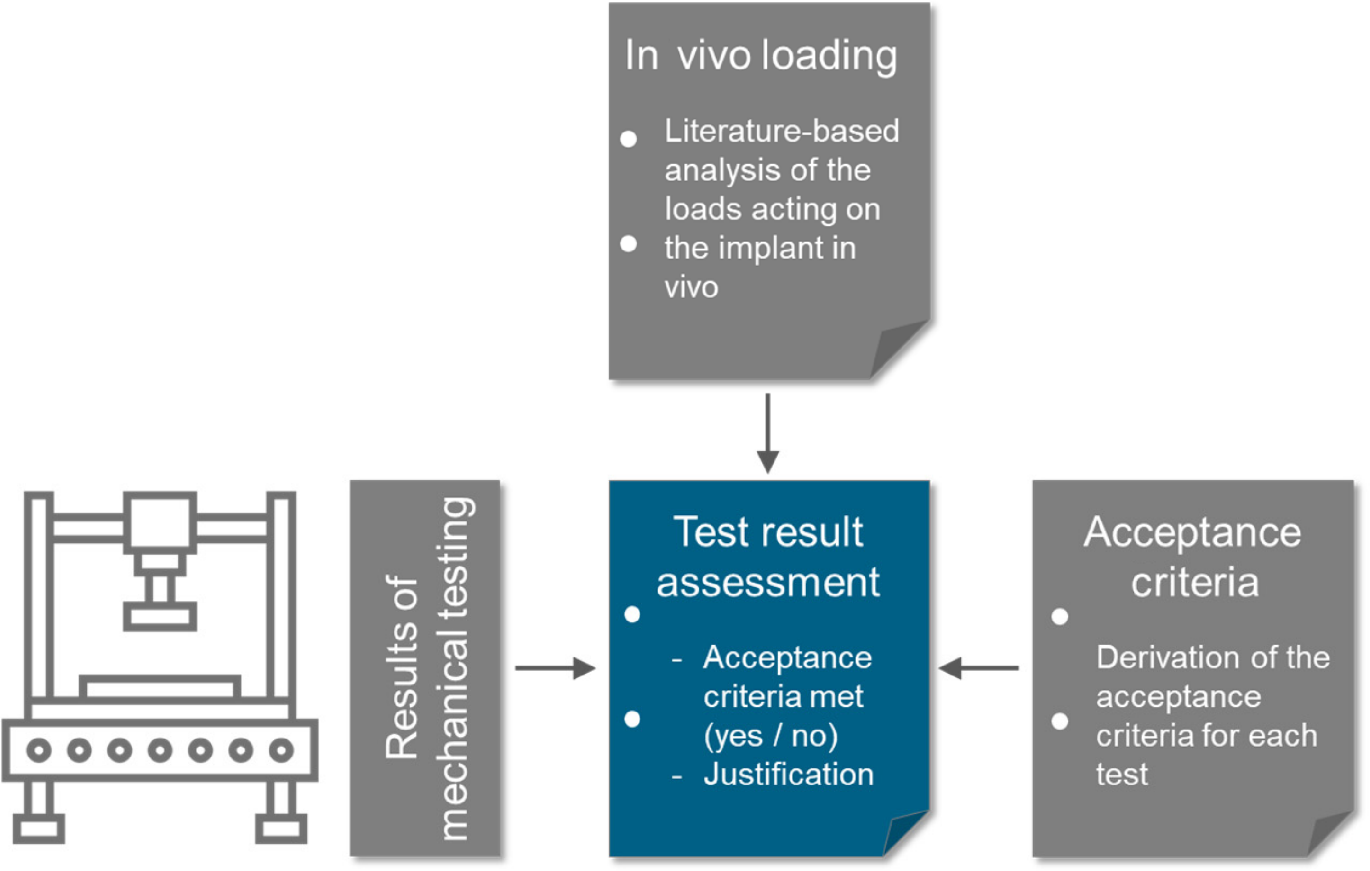
Step 5 of the literature‐based approach to proof the mechanical safety of a spinal implant: Assessment of the test results.

For the assessment, the results are compared to the acceptance criteria (Figure 7). In case this comparison results in ‘pass’, assessment is done. In case the acceptance criteria are not met, further actions need to be taken for example re‐interpretation of the test results, optimization of the implant design, restriction of its use to a certain patient population, or an update of the performance claims.

**FIGURE 8:**
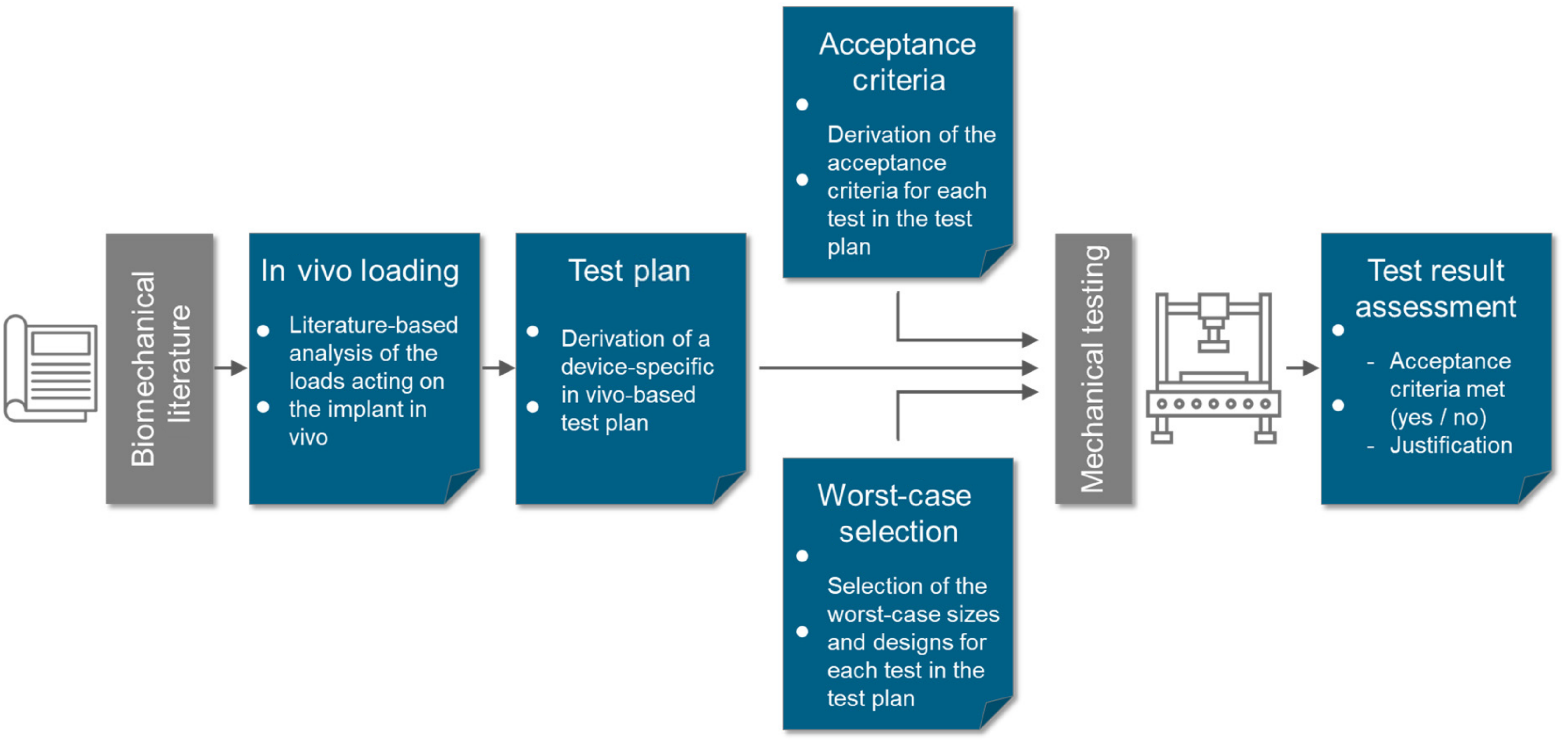
Structure of a literature‐based approach to proof the mechanical safety of a spinal implant.

In case the implant design has to be changed, the literature‐ or risk‐based approach has to be re‐started from step 1 to check whether any revision is required (Figure 8).

## 4 DISCUSSION

The aim of this document was to describe how a risk‐ and literature‐based can support the proof that a spinal implant meets all necessary safety and performance standards. Compared to the conventional approach that uses standard test methods and predicate devices to establish the acceptance criteria, the risk‐ and literature‐based approach demonstrates its strengths especially with novel implants. Novel implants are defined by one or more of the following characteristics:

- New technology
- New design
- New material
- New material combinations
- Extension of indications (also transition areas)
- Extension of target population

In summary, the following steps are taken (Table 4):

- Based on the scientific literature and the implant specifications, the loads acting on the implant in vivo are summarized.
- A test plan is derived from these in vivo loading conditions. In case there is enough evidence from literature that one can argue not to do any testing, this argumentation is to be provided.
- Acceptance criteria are defined for each test described in the test plan based on the in vivo loading of the implant.
- Worst‐case implant sizes and geometries are selected for each test described in the test plan based on FEA or on a theoretical basis.
- Mechanical testing is carried out.
- The test results are assessed basically in terms of a comparison between the results and the acceptance criteria.

This approach aims to demonstrate compliance with Annex II, Chapter 6, and the applicable General Safety and Performance Requirements (GSPRs) in Annex I of the EU Medical Device Regulation (MDR) for the pre‐clinical verification and validation of a novel spinal implant. This comprehensive testing strategy supports the proof that the device meets all necessary safety and performance standards, addressing critical aspects of design verification and validation to prioritize patient safety. By adhering to the regulatory requirements, manufacturers can substantiate the clinical safety and efficacy of their spinal implant, thereby facilitating a smoother path to market access and ensuring ongoing compliance with MDR requirements.

In principle, the scientific literature is the best database that we can use for derivation of an in vivo based mechanical test plan. However, there are certain gaps in literature. Most important for a full chain of arguments is not to leave these gaps open. Mostly, a literature‐based rationale helps to bridge the gap.

As soon as a full chain of arguments is successfully built up, this procedure allows for individual testing, individual test result interpretation and, which is most important, for addressing the real loading that is expected to act on the implant in vivo. This has the potential to further reduce the risk of implant failure.

**FIGURE 9:**
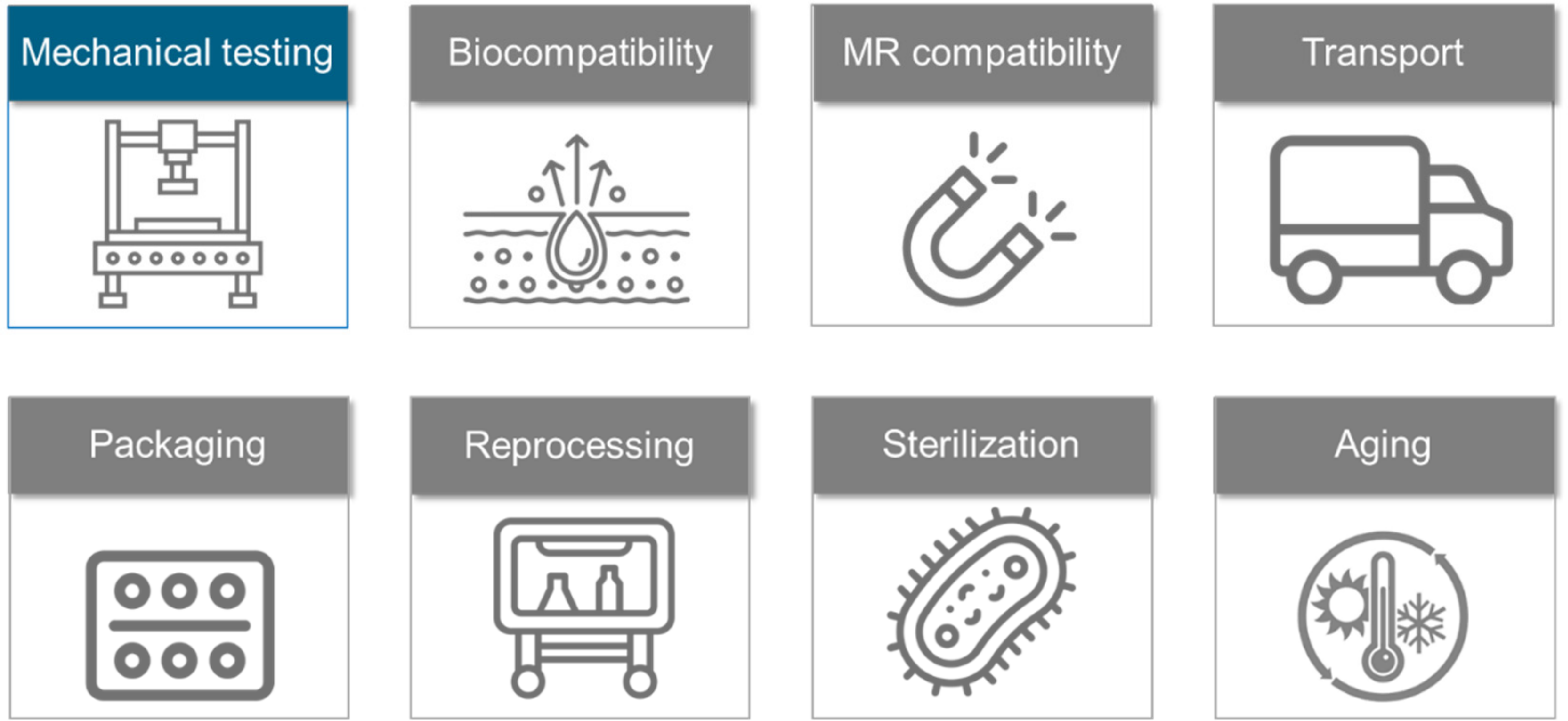
Mechanical testing must be accompanied by safety considerations in various other areas (representative topics).

The primary focus of this paper is on mechanical testing. However, other safety‐related aspects are equally important and must also be taken into account (Figure 9). Some of these aspects can be integrated into mechanical testing, such as aging (mechanical testing following aging), reprocessing (mechanical testing after reprocessing), or transport validation.

In contrast, other safety concerns, such as biocompatibility or MR‐compatibility, require distinct and separate testing procedures. These two concerns mainly apply to implants made of a novel material:

Traditional materials such as titanium and polyetheretherketone (PEEK) are the most widely used materials in spinal implants, particularly for components like screws, cages, and rods. Titanium is renowned for its strength, corrosion resistance, and biocompatibility, whereas PEEK may be favored due to its flexibility and radiolucency (compatibility with imaging techniques). PEEK has a Young's modulus that is more similar to bone, enhancing load sharing, but its osteointegrative limitations can result in complications such as pseudoarthrosis.^50^, ^51^ To combine the advantages of both materials, cages are on the market that are made of PEEK but coated with titanium.^52^

Novel and emerging materials mostly aim at enhancing osteointegration and minimizing implant failure. Bioactive glass (claimed to promote bone healing through ion exchange with body fluids), silicon nitride (highlighted for its biocompatibility, wear resistance, and anti‐inflammatory properties) and tantalum (known for its low elastic modulus and enhanced osteointegration) are among these emerging materials.^50^, ^51^, ^53^

In spinal surgery, biologic materials like collagen, hydrogels, autografts and allografts, growth factors or mesenchymal stem cells play a crucial role in promoting healing, enhancing spinal fusion, and supporting tissue regeneration. These biologic materials are chosen based on their biocompatibility, ability to support tissue regeneration, and potential to improve outcomes in spinal surgeries.

For biologic or any other novel material, the regulatory pathway foresees additional steps such as pre‐clinical bench and animal studies (material characterization, biocompatibility testing, sterilization validation, animal studies, and possibly demonstration of clinical performance through trials).

However, these considerations do not impact the mechanical testing processes discussed in this review article.

In conclusion, this review highlights the importance of integrating scientific literature into regulatory practice to optimize patient outcomes and mitigate complications. The literature‐ or risk‐based approach to pre‐clinical mechanical testing provides a robust framework for developing a well‐structured and focused chain of arguments from literature review through testing to the interpretation of test results. This approach has the potential to significantly enhance the efficiency of testing processes, reduce the risk of failure, and mitigate unnecessary patient suffering and healthcare expenses.

By combining scientific biomechanical and clinical knowledge with regulatory guidelines, this review aims to provide valuable insights for clinicians and researchers, with the goal of enhancing patient care and driving innovation in medical device technologies. Through this integration, the review seeks to bridge the gap between scientific advancements and practical applications, fostering improvements in both clinical outcomes and the development of safer, more effective medical devices.
